# Novel Application of Radotinib for the Treatment of Solid Tumors via Natural Killer Cell Activation

**DOI:** 10.1155/2018/9580561

**Published:** 2018-12-31

**Authors:** Kyung Eun Kim, Sunyoung Park, Soyoung Cheon, Dong Yeon Kim, Dae Jin Cho, Jeong Min Park, Dae Young Hur, Hyun Jeong Park, Daeho Cho

**Affiliations:** ^1^Department of Cosmetic Sciences, Sookmyung Women's University, Chungpa-Dong 2-Ka, Yongsan-ku, Seoul 04310, Republic of Korea; ^2^Institute of Convergence Science, Korea University, Anam-ro 145, Seongbuk-ku, Seoul 02841, Republic of Korea; ^3^Nano-Bio Resources Center, Sookmyung Women's University, Chungpa-Dong 2-Ka, Yongsan-ku, Seoul 04310, Republic of Korea; ^4^Cent'l Res. Inst, Ilyang Pharm. Co. Ltd, Hagal-ro 136beon-gil, Giheung-gu, Yongin-si, Gyeonggi-do 17096, Republic of Korea; ^5^Department of Anatomy, Inje University College of Medicine, Busan 47392, Republic of Korea; ^6^Department of Dermatology, Yeouido St. Mary's Hospital, The Catholic University of Korea, Seoul 07345, Republic of Korea

## Abstract

Radotinib (Supect™) was developed to treat chronic myeloid leukemia (CML) as a BCR-ABL1 tyrosine kinase inhibitor (TKI). Other TKIs, including imatinib and nilotinib, were also developed for treatment of CML, and recent studies were increasing about the therapeutic effects of other TKIs on solid tumors. However, the effect of radotinib on solid tumors has not yet been investigated. In this study, radotinib killed CML cell line K562 directly; however, radotinib did not enhance NK cell cytotoxicity against K562 cells. Because K562 is known as a Fas-negative cell line, we investigated whether radotinib could regulate cell cytotoxicity against various Fas-expressing solid cancer cell lines. Radotinib dramatically increased NK cell cytotoxicity against various Fas-expressing solid cancer cells, including lung, breast, and melanoma cells. Additionally, the efficiency of radotinib-enhanced cytotoxicity was lower in Fas siRNA-transfected cells than in negative controls, suggesting that Fas signaling might be involved in the radotinib-enhanced NK cell cytotoxicity. This study provides the first evidence that radotinib could be used as an effective and strong therapeutic to treat solid tumors via upregulation of NK cell cytotoxicity, suggesting that radotinib has indirect killing mechanisms via upregulation of antitumor innate immune responses as well as direct killing activities for CML cells.

## 1. Introduction

Radotinib (Supect™; C_27_H_21_F_3_N_8_OHCl, 4-methyl-N-[3-(4-methyl-imidazol-1-yl)-5-trifluoromethyl-phenyl]-3-(4-pyrazin-2-yl-pyrimidin-2-ylamino)-benzamide hydrochloride) was developed to treat chronic myeloid leukemia (CML) as a breakpoint cluster region-Abelson (BCR-ABL) 1 tyrosine kinase inhibitor (TKI). Radotinib is approved as a second-line treatment for CML in South Korea [1]. The structure of radotinib is very similar to that of other BCR-ABL inhibitors, including imatinib (first-line treatment) and nilotinib (second-line treatment).

Several studies have reported the effect of imatinib and/or nilotinib on natural killer (NK) cell activity [2–4]. Imatinib specifically at all treatment concentrations does not affect interferon- (IFN-) *γ* production or NK cell cytotoxicity in the CML cell line, K562. However, high concentrations of nilotinib inhibit IFN-*γ* production, resulting in decreased NK cell activity [2]. Nilotinib induces cell death of CD56^bright^CD16^−^ NK cells, well-known as a cytokine-producing NK cell subset. These results suggest that the suppression of IFN-*γ* production by nilotinib is due to cytokine-producing NK cell death [2]. Additionally, imatinib and nilotinib do not regulate granule expression, such as perforin [3]. Other study has suggested that imatinib and nilotinib do not affect activating or inhibitory receptors on NK cells; however, chemokine receptor CXCR4 are increased by treatment of imatinib or nilotinib, suggesting the off-target effects of TKIs on immune cells [4].

NK cells, CD56^+^CD3^−^ cytotoxic lymphocytes in the blood, play a critical role in the innate immune system through spontaneous elimination of cancerous and virus-infected cells. The cytolytic activity of NK cells is mediated by Fas/Fas ligand interaction, granule exocytosis, and antibody-dependent cell-mediated cytotoxicity [5]. Fas is part of a death receptor containing a conserved death domain in its intracytoplasmic domain. Activated NK cells express Fas ligand and recognize Fas-expressing target cells via Fas/Fas ligand interaction. This interaction leads to activation of a caspase cascade and ultimately apoptotic mechanisms in target cells [6, 7].

Although other TKIs, such as imatinib and nilotinib, do not enhance NK cell activity, the effect of radotinib on NK cell cytotoxicity has not been investigated. In this study, we demonstrate anticancer effects of radotinib via upregulation of NK cell cytotoxicity against Fas-expressing cancer cells.

## 2. Materials and Methods

### 2.1. Cell Culture and siRNA Transfection

The human CML cell line K562, human lung carcinoma cell lines A549 and NCI-H460, human melanoma cell lines A375 and SK-MEL-5, and human breast cancer cell lines MDA-MB-231 and MCF-7 were purchased from ATCC (Manassas, VA, USA). K562 cells were cultured in a RPMI-1640 medium (Gibco), and other cells were cultured in Dulbecco's Modified Eagle Medium. Both media were supplemented with 2 mM L-glutamine, 100 U/ml penicillin, 100 mg/ml streptomycin, and 10% heat-inactivated fetal bovine serum. Cells were maintained in a 5% CO_2_ incubator at 37°C.

At approximately 70% confluency, A549 cells were transfected with 50 pmole Fas siRNA using Lipofectamine RNAiMAX (Invitrogen, Carlsbad, CA, USA) per manufacturer's instructions. Commercially available human Fas siRNA and negative control siRNA were purchased from Santa Cruz Biotechnology (Santa Cruz Biotechnology, CA, USA). Transfection efficiency was confirmed by surface staining analysis using a FACSCalibur (BD Biosciences, San Jose, CA, USA) using phycoerythrin- (PE-) conjugated Fas antibody (BD Biosciences) or PE-conjugated mouse IgG isotype control.

### 2.2. Isolation of Human Peripheral Blood Lymphocytes and NK Cells

Human blood samples were obtained from Inje University Busan Paik Hospital (Korea). All studies using human subjects were approved by the Institutional Review Board (Inje IRB/1). Peripheral blood mononuclear cells (PBMC) were isolated from the blood by density gradient centrifugation using Ficoll-Paque (Sigma, St. Louis, MO, USA), and then peripheral blood lymphocytes (PBLs) were collected after monocyte depletion. Briefly, PBMC were resuspended in a RPMI1640 medium supplemented with 10% fetal bovine serum (FBS), and incubated on plastic culture dishes in 5% CO_2_ incubator at 37°C for overnight. Suspended cells including PBLs were collected. Human primary NK cells were isolated from PBLs using MACS NK cell isolation kit (Miltenyi Biotec, Bergisch Gladbach, Germany) as per the manufacturer's recommendation.

### 2.3. Cytotoxicity Assay

A cytotoxicity assay was performed as previously described [8]. Briefly, effector cells, such as isolated PBLs or purified NK cells, were treated with radotinib at indicated concentrations or with recombinant human interleukin- (IL-) 2 (50 U/ml) for 48 h. Target cells were stained with carboxyfluorescein diacetate succinimidylester (Molecular Probes Inc., USA) for five min at 37°C. After three washes with cold complete medium, the labeled target cells were incubated with effector cells. The assay was performed in triplicate with various effector cell to target cell (E : T) ratios. After incubation at 37°C in 5% CO_2_ for 2 h, the target cell lysis was analyzed by 7-aminoactinomycin D (7-AAD) (BD Biosciences) staining using a FACSCalibur (BD Biosciences) with Cell Quest software.

To block the Fas-Fas ligand interactions, approximately 0.5-2 *μ*g of recombinant human soluble Fas (R&D systems) was incubated with resting or radotinib-treated NK cells before the cytotoxicity assay. After preincubation for 1 h, the cytotoxicity assay was performed as described above without washing.

### 2.4. Statistical Analysis

All values were analyzed with an unpaired Student's *t*-test. Statistical analyses were performed using GraphPad Prism 5 (GraphPad Software, La Jolla, CA, USA).

## 3. Results and Discussion

**Figure 1:**
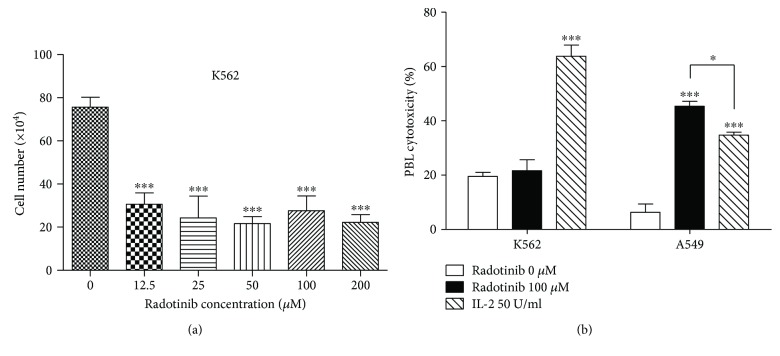
Radotinib enhances cytolytic activity of PBLs against A549 cells, but not K562 cells. (a) A CML cell line, K562, was treated with various concentrations of radotinib for 24 h to determine the direct effect of radotinib on K562 cell death. A trypan blue exclusive assay was performed to count live cells. (b) PBLs were isolated from healthy donors and incubated with 0 or 100 *μ*M radotinib for 48 h. IL-2 was used as a positive control. To perform the cytotoxicity assay, K562 or A549 cells were labeled with carboxyfluorescein succinimidyl ester (CFSE) and then used as target cells. Radotinib-stimulated PBLs were incubated with CFSE-labeled target cells for 2 h (E : T ratio = 5 : 1) to measure the cytolytic activity of PBLs. After incubation, cells were stained with 7-AAD, and FACSCalibur was used to analyze CFSE and 7-AAD double-positive target cells. Data are reported as mean ± SD. All values were analyzed by unpaired Student's *t*-tests using GraphPad Prism 5. ^∗^ *P* < 0.05 and ^∗∗∗^ *P* < 0.001. All data presented are representative of three independent experiments.

Radotinib, a novel BCR-ABL1 TKI, is an effective therapeutic drug for treating CML [1]. BCR-ABL1 fusion is the main pathogenesis of CML, leading to uncontrolled proliferation. Therefore, TKIs targeting BCR-ABL1 have been developed as therapeutics for CML because of their inhibition of uncontrolled proliferation. To confirm the effect of radotinib on CML cells, we used a representative CML cell line, K562. Cell viability of K562 cells was significantly reduced even at the lowest concentration of radotinib (12.5 *μ*M), indicating that radotinib kills K562 cells directly (Figure 1(a)). It has been reported that another standard TKI, imatinib, inhibits cancer cell proliferation via the regulation of cell cycle [9]. Therefore, our data suggest that radotinib is able to inhibit cell proliferation and induce cell apoptosis by inhibiting BCR-ABL1 as well as imatinib.

**Figure 2:**
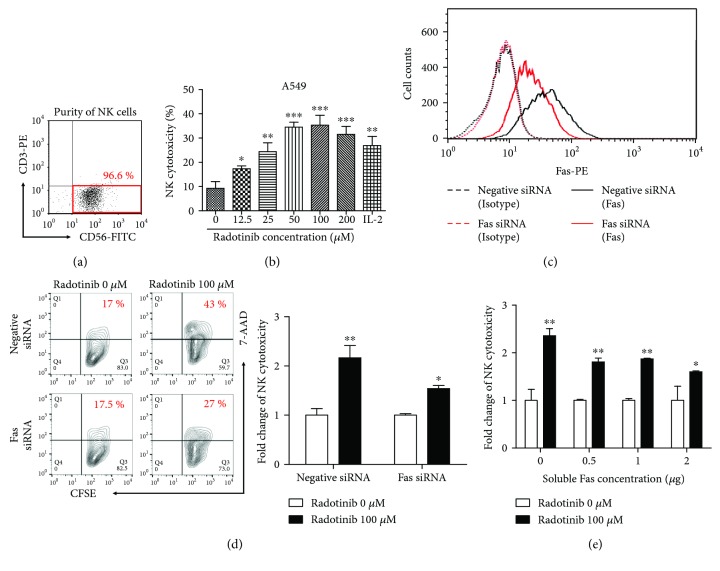
Radotinib enhances cytolytic activity of NK cells against Fas-expressing A549 cells. (a) Primary NK cells were isolated from healthy donors to perform NK cell cytotoxicity assay. The purity of CD3^−^CD56^+^ NK cells was 96.6%. (b) To determine the effect of radotinib on the cytolytic activity of NK cells against A549 cells, the cells were treated with various concentrations of radotinib (0, 12.5, 25, 50, 100, and 200 *μ*M) for 48 h and the cytotoxicity assay was performed (E : T ratio = 5 : 1). (c) To determine if the effect of radotinib on NK cytotoxicity was mediated by the Fas receptor, Fas expression was transiently downregulated by Fas siRNA transfection into A549 cells. At approximately 70% confluency, A549 cells were incubated with 50 pmole Fas-specific siRNA or negative control siRNA using Lipofectamine RNAiMAX. Surface expression of Fas on A549 cells was determined by staining with PE-conjugated Fas antibody (solid line). PE-conjugated mouse IgG antibody was used as an isotype control (dotted line). (d) The effect of radotinib on NK cytolytic activity against Fas siRNA-transfected A549 cells was determined by cytotoxicity assay. Radotinib-treated NK cells were used as effector cells, and Fas siRNA-transfected A549 cells or control cells were used as target cells (E : T ratio = 2 : 1). All values were normalized relative to the control (radotinib 0 *μ*M). The relative level was set to 1 for the control. (e) To further confirm the involvement of Fas-Fas ligand interaction in the radotinib-enhanced NK cytotoxicity, recombinant human soluble Fas was used to block Fas ligand on NK cells. Various concentrations of soluble Fas were preincubated with resting NK cells or radotinib-treated NK cells for 1 h, and then cytotoxicity assays were performed (E : T ratio = 2 : 1). All values were normalized relative to the control (radotinib 0 *μ*M). The relative level was set to 1 for the control. Data are reported as mean ± SD. All values were analyzed by unpaired Student's *t*-tests using GraphPad Prism 5. ^∗^ *P* < 0.05, ^∗∗^ *P* < 0.01, and ^∗∗∗^ *P* < 0.001. All data presented are representative of three independent experiments.

To determine the ability of radotinib to kill K562 cells via the cytolytic activity of peripheral blood lymphocytes (PBLs), we performed a cytotoxicity assay using radotinib-treated PBLs as effector cells and K562 cells as target cells. Although radotinib directly and effectively killed K562 cells, it did not enhance the cytolytic activity of PBLs against K562, whereas IL-2 significantly stimulated cytotoxicity of PBLs (Figure 1(b)). Because K562 cells are Fas-negative cells [10–12], we hypothesized that radotinib may regulate cell cytotoxicity against certain types of tumor cells, such as Fas-expressing cells. To confirm the effect of radotinib on the cytotoxicity of PBLs against Fas-expressing cells, we determined the Fas expression in A549 cell lines. As shown in Figure 2(b), A549 cells highly expressed the Fas receptor. Consistent with these differences in Fas expression, radotinib dramatically increased the cytolytic activity of PBLs only in A549 cells (Figure 1(b)) suggesting a novel therapeutic effect of radotinib on solid tumor other than CML.

We next isolated human primary NK cells from PBLs to determine whether the cytolytic activity of PBLs was mediated by NK cell cytotoxicity. The purified NK cells (purity = 96.6% as shown in Figure 1(a)) were incubated with various doses (0, 12.5, 25, 50, 100, and 200 *μ*M) of radotinib, which did not affect the viability of NK cells (data not shown). NK cell cytotoxicity was markedly upregulated upon radotinib treatment. This significant increase in cytotoxicity with radotinib treatment was dose-dependent (Figure 2(b)). To determine the involvement of Fas-FasL interaction in radotinib-enhanced NK cell cytotoxicity, the A549 cells were transiently transfected with Fas-specific siRNA or negative control siRNA. Surface staining and flow cytometry were then performed to detect Fas expression on the transfected cells. As shown in Figure 2(c), surface expression of Fas decreased in the A549 cells transfected with Fas-specific siRNA compared to controls.  Figure 2(d) shows that the efficiency of radotinib-enhanced cytotoxicity was lower in the Fas-specific siRNA-transfected A549 cells than in the control cells. To further confirm this result, recombinant human soluble Fas was used to block Fas-FasL interaction. The soluble Fas was preincubated with NK cells to bind Fas ligands on their surface before the cytotoxicity assay.  Figure 2(e) shows that the efficiency of radotinib-enhanced cytotoxicity was significantly decreased by the preincubation with soluble Fas.

**Figure 3:**
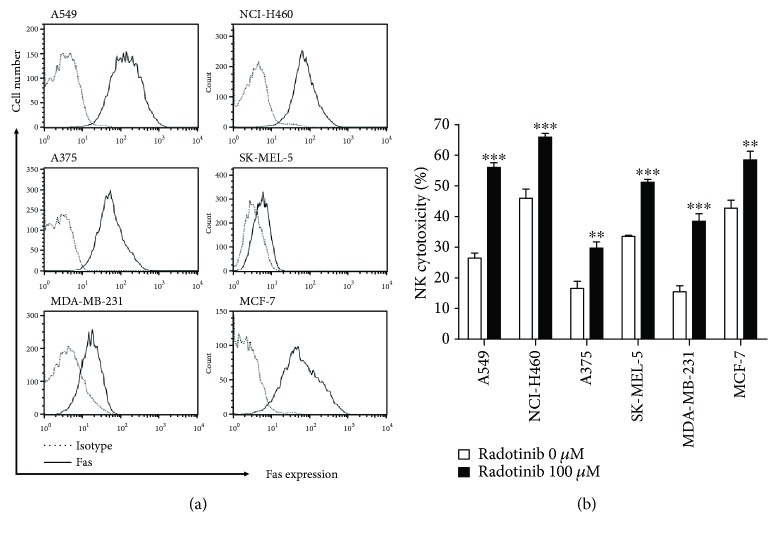
Radotinib enhances cytolytic activity of NK cells against various cancer cells expressing Fas receptor. To further explore the involvement of the Fas receptor in cytolysis, various cancer cell lines were used to analyze the radotinib-enhanced cytolytic activity of NK cells. (a) Surface expression of Fas on various cancer cells, including A549, NCI-H460, A375, SK-MEL-5, MDA-MB-231, and MCF-7, was determined by staining with PE-conjugated Fas antibody (solid line). PE-conjugated mouse IgG antibody was used as an isotype control (dotted line). (b) The effect of radotinib on NK cytolytic activity against various cancer cell lines was determined by cytotoxicity assays. Radotinib-treated NK cells were used as effector cells, and cancer cell lines were used as target cells (E : T ratio = 2 : 1). Data are reported as mean ± SD. All values were analyzed by unpaired Student's *t*-tests using GraphPad Prism 5. ^∗∗^ *P* < 0.01 and ^∗∗∗^ *P* < 0.001. All data presented are representative of three independent experiments.

Additionally, the expression of Fas ligand on NK cells was confirmed to determine the effect of radotinib on Fas ligand expression of NK cells. Fas ligand expression was slightly enhanced by radotinib treatment on NK cells. Primary NK cells were isolated from nine healthy donors and then incubated with 100 *μ*M of radotinib for 48 h. The average of mean fluorescence intensity (MFI) of Fas ligand expression in all nine samples increased about 10~20% as shown in Supplementary Figure S1. Fas ligand expression on NK cells tended to be increased by radotinib treatment; however, this increase has been shown in part of the donors (3 individuals among 9 donors). Nevertheless, it is certain that radotinib significantly enhances NK cell cytotoxicity against various solid cancer cell lines even in donors whose Fas ligand does not increase. NK cell cytotoxicity of radotinib in the other Fas-expressing cell lines was tested by using a different lung cancer cell line, NCI-H460; the melanoma cell lines, SK-MEL-5 and A375; and the breast cancer cell lines MCF-7 and MDA-MB-231. These cell lines were used as target cells in which Fas expression was confirmed by flow cytometry (Figure 3(a)).  Figure 3(b) shows that radotinib-stimulated NK cells effectively killed all the solid tumor cells tested.

Thus, it could be a difference by NK subsets in the individuals or the other NK-activating receptors. Several studies have reported various NK cell subsets, such as CD56^bright^ or CD56^dim^. It is well-known that CD56^dim^/CD16^+^ NK cells are the major population in the peripheral blood having cytolytic activity [13, 14]. Especially, it was reported that Fas ligand-mediated NK cell cytotoxicity depends on the expression of CD2 on CD56^dim^ NK cells. CD2^+^CD56^dim^ NK subsets highly express Fas ligand, implying that the degree of Fas ligand expression is different in the subdivided NK subsets [15]. Additionally, 2B4 and LFA-1 also upregulate Fas ligand expression on NK cells while CD94/NKG2A inhibits Fas ligand-mediated cytotoxicity [16]. Therefore, further study will be required to demonstrate the effect of radotinib on Fas ligand expression by subdividing the NK subset in the individuals for understanding radotinib-enhanced NK cell killing activity to design future clinical application for therapy.

In addition to Fas-FasL interaction, it is known that NK cell activation is regulated by the balance of activating and inhibitory receptors; the expression of several receptors on NK cells was determined, as shown in Supplementary Figure S2. Both activating (NKp46, NKp44, NKp80, and NKG2D) and inhibitory (CD94, KIR2DL1, KIR2DL2/DL3, and KIR3DL2) receptors were not affected by treatment with radotinib. A recent study has investigated the effect of other TKIs including imatinib and nilotinib, resulting that both TKIs also do not enhance activating or inhibitory receptors on NK cells. However, these TKIs increase the chemokine receptor CXCR4 on the surface of NK cells and monocytes [4]. Thus, it is needed to investigate the effect of radotinib on the expression of chemokine receptors or adhesion molecules.

**Figure 4:**
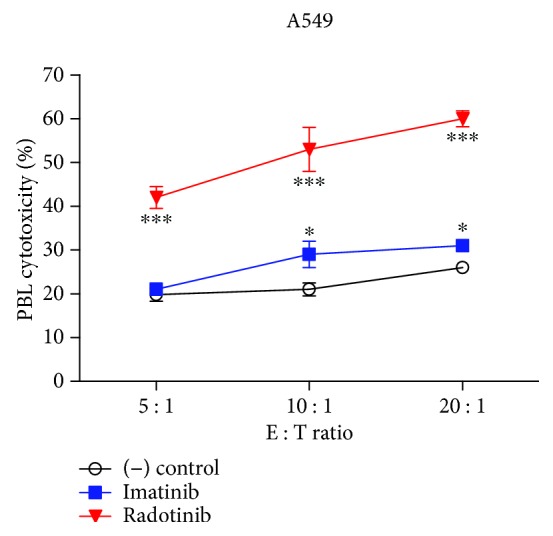
A comparison of the cytolytic activity of radotinib and imatinib mesylate. A comparison of the cytolytic activity of radotinib and imatinib mesylate, a TKI for treating CML, against Fas-expressing A549 cells was performed using radotinib- or imatinib mesylate-treated PBLs as effector cells and A549 cells as target cells in a cytotoxicity assay. Data are reported as mean ± SD. All values were analyzed by unpaired Student's *t*-tests using GraphPad Prism 5. ^∗^ *P* < 0.05 and ^∗∗∗^ *P* < 0.001. All data presented are representative of three independent experiments.

Here, we confirmed that radotinib enhanced NK cell cytotoxicity against Fas-expressing cancer cells, but not CML cell line K562, providing new insight into the mechanisms of the antitumor effects of radotinib against solid tumors. These results, indicating that radotinib does not induce K562 cell death through PBL-mediated cytotoxicity, are comparable to those of other TKIs, including imatinib and nilotinib, that do not enhance the cytolytic activity of PBLs against K562 cells [2]. Therefore, in order to examine whether imatinib can also kill Fas-expressing cancer cells mediated by PBL cytotoxicity, PBLs were treated with imatinib mesylate (100 *μ*M; Sigma-Aldrich) to enhance cytolytic activity. Cytotoxicity against A549 cells increased slightly upon imatinib mesylate treatment (Figure 4). However, enhancement of cytolytic activity upon radotinib treatment was greater.

In conclusion, this study provides the first evidence that radotinib, which was developed as a therapeutic drug for CML, can be used for a novel application for treatment of solid tumors. This study shows a strong evidence that radotinib enhances NK cytolytic activity, resulting in anticancer effects against various solid cancer cell lines. It suggests that radotinib has indirect killing mechanisms via upregulation of antitumor innate immune responses as well as direct killing activities for BCR-ABL1-positive CML cells. These off-target effects of radotinib in immune cells indicate that radotinib could be the most effective and strongest therapeutics for solid tumors. Additionally, radotinib has been approved and currently used for treating CML patients and has these off-target effects without killing of normal immune cells as shown in our study. Therefore, we expect to be able to apply it directly to the treatment of other solid cancers.

## Data Availability

The data used to support the findings of this study are included within the article.

## Supplementary Materials

Supplementary Materials Supplementary Figure 1: effect of radotinib on surface expression of Fas ligand in primary NK cells. To determine the effect of radotinib on Fas ligand expression of NK cells, primary NK cells were isolated from nine healthy donors and then incubated with 100 *μ*M of radotinib for 48 h. (A) Surface expression of Fas ligand on NK cells was determined by staining with PE-conjugated Fas ligand antibody (solid line). PE-conjugated mouse IgG antibody was used as an isotype control (dotted line). Data presented is a representative of three donors showing increased expression of Fas ligand. (B) The average of mean fluorescence intensity (MFI) of Fas ligand expression in all nine samples was shown. All values were normalized relative to the control (radotinib 0 *μ*M). The relative level was set to 1 for the control. Supplementary Figure 2: effect of radotinib on the expression of activating or inhibitory receptors. To determine the effect of radotinib on activating or inhibitory receptors of NK cells, surface FACS staining was performed as described in Materials and Methods. Resting NK cells or radotinib-treated NK cells were incubated with antibodies as follows: PE-conjugated NKp44, PE-conjugated NKp46, PE-conjugated NKp80, PE-conjugated NKG2D, FITC-conjugated CD94, APC-conjugated KIR2DL1, PE-conjugated KIR2DL2/DL3, PE-conjugated KIR3DL2 (solid line), or isotype controls (dotted line). The expression level of surface molecules was detected using FACSCalibur (BD Biosciences); then data was analyzed using FlowJo software. All data presented are representative of three independent experiments.
