# Supplementary material for: Novel Application of Radotinib for the Treatment of Solid Tumors via Natural Killer Cell Activation
Source: J Immunol Res. 2018 Dec 31;2018:9580561. doi: 10.1155/2018/9580561 (PMC6330826; doi:10.1155/2018/9580561)
Supplement: Supplementary Materials — Supplementary Figure 1: effect of radotinib on surface expression of Fas ligand in primary NK cells. To determine the effect of radotinib on Fas ligand expression of NK cells, primary NK cells were isolated from nine healthy donors and then incubated with 100 μM of radotinib for 48 h. (A) Surface expression of Fas ligand on NK cells was determined by staining with PE-conjugated Fas ligand antibody (solid line). PE-conjugated mouse IgG antibody was used as an isotype control (dotted line). Data presented is a representative of three donors showing increased expression of Fas ligand. (B) The average of mean fluorescence intensity (MFI) of Fas ligand expression in all nine samples was shown. All values were normalized relative to the control (radotinib 0 μM). The relative level was set to 1 for the control. Supplementary Figure 2: effect of radotinib on the expression of activating or inhibitory receptors. To determine the effect of radotinib on activating or inhibitory receptors of NK cells, surface FACS staining was performed as described in Materials and Methods. Resting NK cells or radotinib-treated NK cells were incubated with antibodies as follows: PE-conjugated NKp44, PE-conjugated NKp46, PE-conjugated NKp80, PE-conjugated NKG2D, FITC-conjugated CD94, APC-conjugated KIR2DL1, PE-conjugated KIR2DL2/DL3, PE-conjugated KIR3DL2 (solid line), or isotype controls (dotted line). The expression level of surface molecules was detected using FACSCalibur (BD Biosciences); then data was analyzed using FlowJo software. All data presented are representative of three independent experiments. [file 9580561.f1.pdf]

# Supplementary Materials and Methods

## Expression of surface molecules on NK cells

To determine the effect of radtinib on various surface molecules of NK cells, including Fas ligand, activating receptors, and inhibitory receptors, surface FACS staining was performed. Equal numbers of the cells ($1 \times 10^6$) were transferred into tubes. After washing twice with PBS, cells were incubated with the following antibodies: PE-conjugated Fas ligand, FITC-conjugated CD56, APC-conjugated CD16, PE-conjugated NKp44, PE-conjugated NKp46, PE-conjugated NKG2D, FITC-conjugated CD94, APC-conjugated KIR2DL1, PE-conjugated KIR2DL2/DL3 (BD Biosciences), PE-conjugated NKp80, PE-conjugated KIR3DL2 (R&D Systems), or isotype controls. After incubation for 30 min on ice, stained cells were washed twice with PBS. The expression level of surface molecules was detected using a FACSCalibur (BD Biosciences), and then data was analyzed using FlowJo software.

# Supplementary Figure and Figure Legends

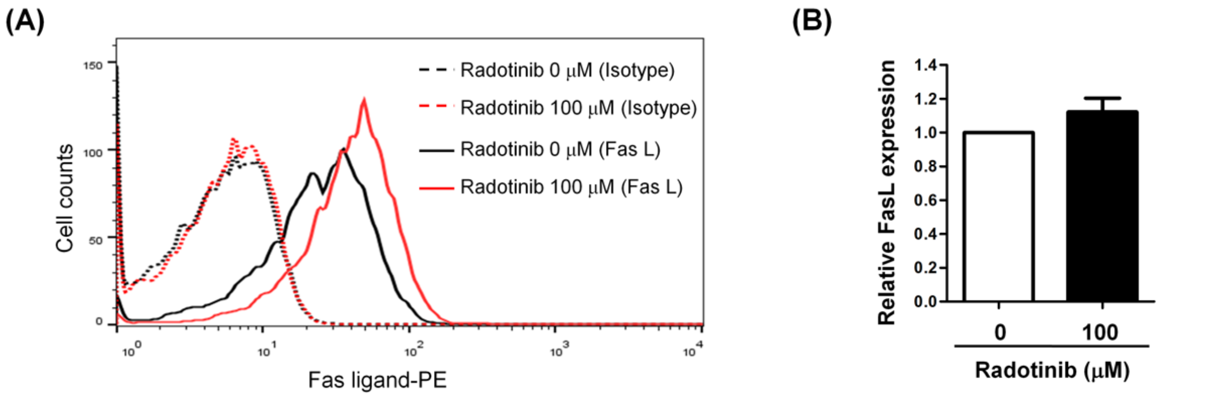


**Supplementary Figure 1. Effect of Radotinib on surface expression of Fas ligand in primary NK cells**

To determine the effect of radotinib on Fas ligand expression of NK cells, primary NK cells were isolated from nine healthy donors, and then incubated with 100 μM of radotinib for 48 hr. (A) Surface expression of Fas ligand on NK cells was determined by staining with PE-conjugated Fas ligand antibody (solid line). PE-conjugated mouse IgG antibody was used as an isotype control (dotted line). Data presented is a representative of three donors showing increased expression of Fas ligand. (B) The average of mean fluorescence intensity (MFI) of Fas ligand expression in all nine samples was shown. All values were normalized relative to the control (radotinib 0 μM). The relative level was set to 1 for the control.

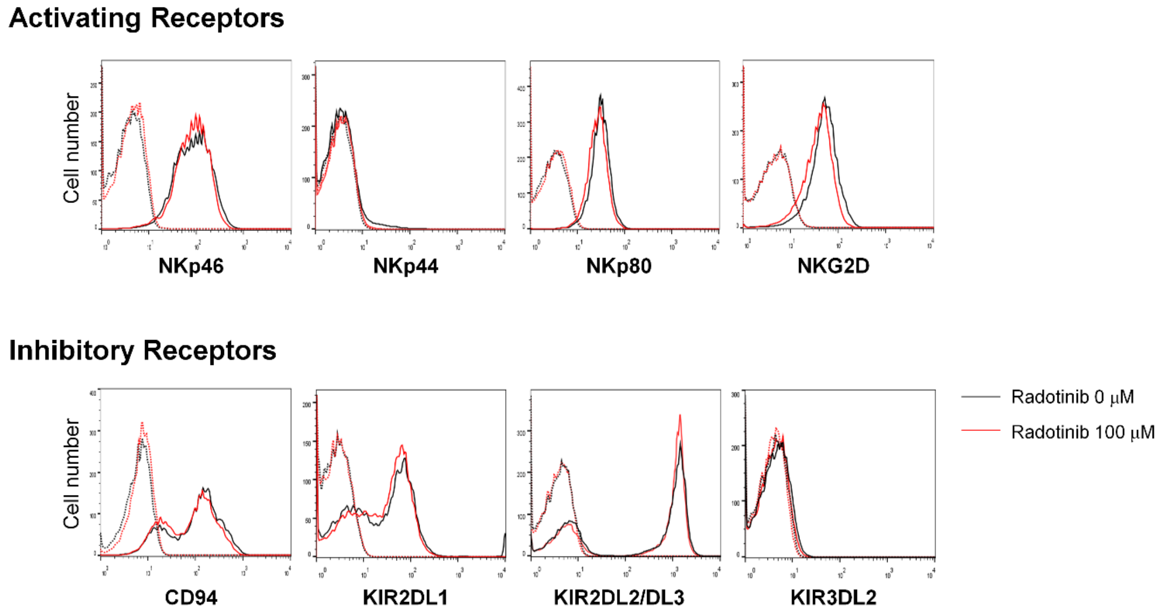


**Supplementary Figure 2. Effect of Radotinib on the expression of activating or inhibitory receptors**

To determine the effect of radotinib on activating or inhibitory receptors of NK cells, surface FACS staining was performed as described in the Materials and Methods. Resting NK cells or radotinib-treated NK cells were incubated with antibodies as following: PE-conjugated NKp44, PE-conjugated NKp46, PE-conjugated NKp80, PE-conjugated NKG2D, FITC-conjugated CD94, APC-conjugated KIR2DL1, PE-conjugated KIR2DL2/DL3, PE-conjugated KIR3DL2 (solid line), or isotype controls (dotted line). The expression level of surface molecules was detected using FACSCalibur (BD Biosciences), then data was analyzed using FlowJo software. All data presented are representative of three independent experiments.
